# Characterization of *Chlamydomonas reinhardtii* Mutants That Exhibit Strong Positive Phototaxis

**DOI:** 10.3390/plants10071483

**Published:** 2021-07-20

**Authors:** Jun Morishita, Ryutaro Tokutsu, Jun Minagawa, Toru Hisabori, Ken-ichi Wakabayashi

**Affiliations:** 1 Laboratory for Chemistry and Life Science, Institute of Innovative Research, Tokyo Institute of Technology, Yokohama 226-8503, Japan; senxiachunfalilv@gmail.com (J.M.); thisabor@res.titech.ac.jp (T.H.); 2 School of Life Science and Technology, Tokyo Institute of Technology, Yokohama 226-8503, Japan; 3 Division of Environmental Photobiology, National Institute for Basic Biology, Okazaki 444-8585, Japan; tokutsu.ryutaro.4e@kyoto-u.ac.jp (R.T.); minagawa@nibb.ac.jp (J.M.); 4 Department of Basic Biology, Faculty of Life Science, The Graduate University for Advanced Studies, SOKENDAI, Okazaki 444-8585, Japan

**Keywords:** *Chlamydomonas*, phototaxis, photosynthesis, photoprotection

## Abstract

The most motile phototrophic organisms exhibit photo-induced behavioral responses (photobehavior) to inhabit better light conditions for photosynthesis. The unicellular green alga *Chlamydomonas reinhardtii* is an excellent model organism to study photobehavior. Several years ago, we found that *C. reinhardtii* cells reverse their phototactic signs (i.e., positive and negative phototaxis) depending on the amount of reactive oxygen species (ROS) accumulated in the cell. However, its molecular mechanism is unclear. In this study, we isolated seven mutants showing positive phototaxis, even after the induction of negative phototaxis (*ap1~7*: always positive) to understand the ROS-dependent regulatory mechanism for the phototactic sign. We found no common feature in the mutants regarding their growth, high-light tolerance, and photosynthetic phenotypes. Interestingly, five of them grew faster than the wild type. These data suggest that the ROS-dependent regulation of the phototactic sign is not a single pathway and is affected by various cellular factors. Additionally, the isolation and analyses of mutants with defects in phototactic-sign regulation may provide clues for their application to the efficient cultivation of algae.

## 1. Introduction

For motile phototrophic organisms, photo-induced behavioral responses (photobehavior) are important to maintain optimal light exposure for their photosynthetic activities. The unicellular green alga *Chlamydomonas reinhardtii* is an ideal model organism for studying photo responses because it exhibits striking photobehavior immediately after photoreception [1,2]. The typical photobehavior in *C. reinhardtii* includes phototaxis and photoshock response. Phototaxis is a behavior in which cells swim toward or away from incident light (called positive or negative phototaxis, respectively). Photoshock response is a behavior in which cells stop swimming or swim backward for a short period after a sudden change in light intensity.

Both behaviors are regulated by the following two organelles: the eyespot and cilia. The eyespot is a directional photoreceptive organelle [3,4]. The eyespot constitutes two parts, namely, the carotenoid-granule layers (CGLs) and the photoreceptor protein channel rhodopsins (ChRs). The CGLs function as a quarter-wave stack and reflect light [5,6,7]. These layers line ChRs in the plasma membrane. ChRs function as cation channels upon photoreception [8,9,10]. Due to the light reflection at the CGLs, ChRs sense light only when illuminated from the eyespot side. Cellular Ca^2+^ concentration is suggested to be modulated by photoreception. Ca^2+^-sensitivities of the two cilia are distinct: the cis-cilium, closest to the eyespot, beats stronger than the other when [Ca^2+^]_i_ < ~10^−7^ M, and the trans-cilium, the other one, beats stronger than the other when [Ca^2+^]_i_ > ~10^−7^ M [11]. By this regulation, the forces generated by the two cilia become imbalanced, and the cell changes its swimming direction to exhibit phototaxis.

How, then, do cells reverse their phototactic sign (or direction)? Several signals have been reported to regulate the phototactic signs, such as photosynthetic activity, circadian rhythm, and light intensity [12,13,14]. Among these signals, the cellular reactive oxygen species (ROS) level strongly affects the phototactic sign. After treatment with membrane-permeable ROS reagents, cells show positive phototaxis, whereas after treatment with membrane-permeable ROS-scavenging reagents, cells show negative phototaxis [15]. Even the negatively phototactic strain *agg1* (a wild-type strain CC-124) shows positive phototaxis after treatment with ROS, suggesting that the ROS signal can override other effects [16].

However, this ROS-dependent sign switching of phototaxis is contradictory. ROS is a hazardous byproduct of photosynthesis [17]. Light energy higher than the level that saturates photosynthetic reactions produces excess reductive power, producing ROS. ROS damages various essential cellular materials, including proteins and lipids, and thus, phototrophic organisms have different defense mechanisms against ROS [18]. If *C. reinhardtii* cells show positive phototaxis when the cellular ROS amount increases, the light intensity may increase, and more ROS would be produced; this seems like a suicide. Simultaneously, the induction of positive phototaxis by ROS is highly reproducible [7,16,19,20].

The questions that arise are how and why *C. reinhardtii* cells show ROS-dependent positive phototaxis. For answering these questions, forward genetics will be a strong strategy because the molecular basis for this pathway is difficult to predict. This study introduced random mutations to wild-type *C. reinhardtii* to screen for mutants showing positive phototaxis, even after treatment with ROS scavengers that induce negative phototaxis. Phenotypic analyses of the mutants suggested that the molecules affecting the phototaxis pathways vary.

## 2. Results

### 2.1. Mutant Screening for Phototactic Signs

**Figure 1:**
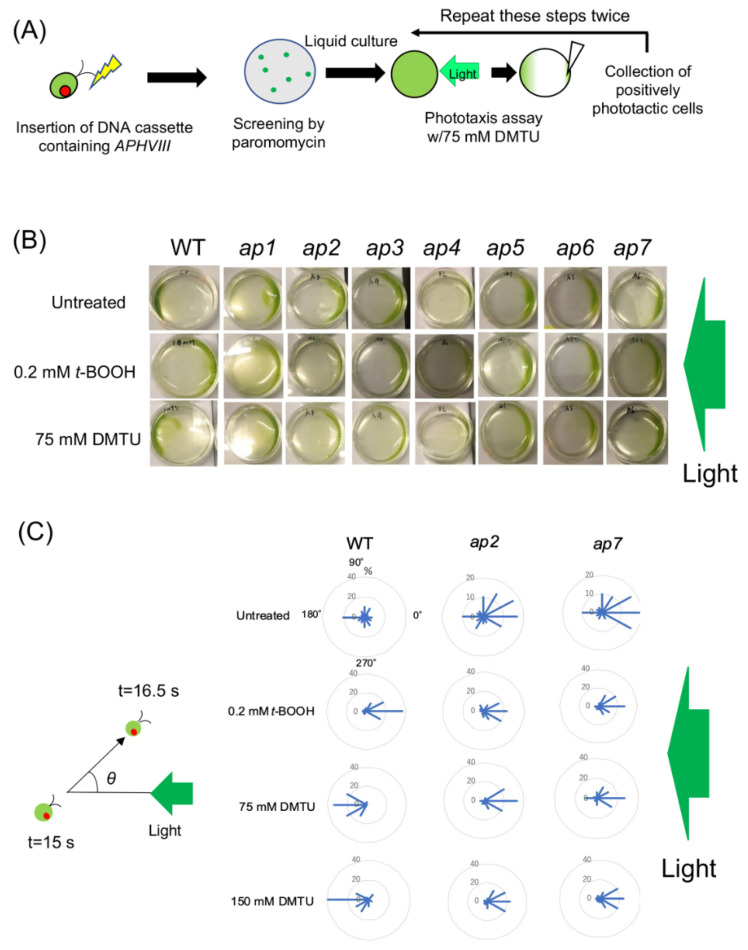
Isolation of *ap* mutants. (**A**) Schematics showing the screening methods for *ap* mutants. After introducing the pSI103-1 vector containing the *APHVIII* gene that confers paromomycin resistance, transformants were inoculated onto the selection agar plate. After collecting colonies, transformant cells (a mutant library) were subjected to phototaxis assay after treatment with 75 mM DMTU that strongly induces negative phototaxis. Cells showing positive phototaxis were collected, cultured again, and subjected to the second phototaxis assay. A mutant constantly showing positive phototaxis was isolated from a library and named *ap* mutant. (**B**) Phototaxis of WT and *ap* mutant cells. Cells were put in a Petri dish, and green light was illuminated from the right side. WT cells showed negative phototaxis without any treatment, positive phototaxis after treatment with 0.2 mM *t*-BOOH, and negative phototaxis after treatment with DMTU. The *ap* mutants showed positive phototaxis in any condition. (**C**) (Left) Schematic of cell-level phototaxis assay. Cell swimming angle (θ) was measured for 1.5 s following 15 s of illumination with a green LED. (Right) Cell-level phototaxis assay of WT and representative *ap* mutants, *ap2* and *ap7*. Polar histograms depicting the percentage of cells moving in a particular direction relative to light illumination from the right (12 bins of 30°; n = 30 cells per condition). Cells were observed after treatment with no reagent (top), 0.2 mM *t*-BOOH to induce positive phototaxis (second from the top), 75 mM (third from the top), or 150 mM DMTU to induce negative phototaxis (bottom).

To generate mutants with defects in the pathways that regulate phototaxis in an ROS-dependent manner, we induced random insertional mutagenesis to wild-type (WT) *C. reinhardtii* using a paromomycin-resistant vector (pSI103-1) [21]. After selection with paromomycin, the mutant library was subjected to phototaxis screening (Figure 1A). Usually, WT cells show negative phototaxis after treatment with membrane-permeable ROS-scavenging reagents such as dimethylthiourea (DMTU) or TEMPOL [15] (Figure 1B,C). We treated the mutagenized cells with DMTU and then screened for mutants showing positive phototaxis (Figure 1A). We repeated this screening twice against seven independent mutant libraries, isolating one mutant from each library that showed positive phototaxis after treatment with DMTU. After backcrossing the mutants with WT twice, we named them *ap* (always positive phototaxis) *1~7*.

On the basis of the results of the phenotypic analysis described later, we selected *ap2* and *ap7* for more detailed phototaxis analysis. In light conditions where the parental WT strain cells tended to show negative phototaxis, both the *ap2* and *ap7* cells showed positive phototaxis. DMTU induced negative and *t*-BOOH, a membrane-permeable ROS reagent, induced positive phototaxis in WT cells (Figure 1C) [15]. DMTU sufficiently induced negative phototaxis at 75 mM in the WT cells, but even after treatment with 150 mM DMTU, the *ap2* and *ap7* cells showed positive phototaxis (Figure 1C).

After the backcross, the phenotypic and genotypic analyses showed that the progenies containing an insertion of the pSI103-1 vector did not always show an *ap* phenotype (Figure S1). These data suggest that the *ap* phenotypes of all seven mutants were caused by the insertion of short DNA fragments derived from the vectors or genomic DNA of dead cells during electroporation. Therefore, the causative genes of *ap1~7* could not be easily traced at this moment.

### 2.2. Morphology and Motility of ap Mutants Are Normal 

To analyze which pathway affects ROS-dependent phototaxis regulation, we examined several phenotypes of *ap* mutants other than phototaxis. First, we tested the morphology and motility of these mutant cells. The cell size (diameter approximated as a sphere), ciliary length, and ciliary beating frequency were measured (Table 1). In each parameter, there was no significant difference between the strains, including WT.

**Figure 2:**
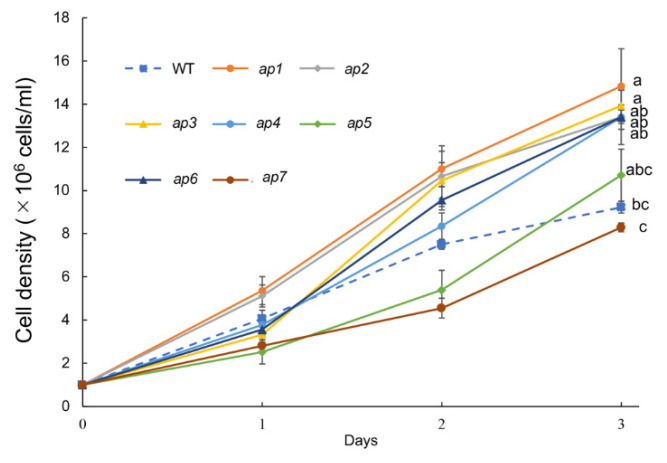
The growth phenotype of *ap* mutants. Precultured cells were collected and suspended in a fresh medium at 1 × 10^6^ cells/mL (day 0) and subjected to culture under 30 μmol photons m^−2^ s^−1^ white light. Cell density was measured every day and means ± S.E.M. (n = 3) are shown. Different letters indicate significant differences (*p* < 0.05, one-way ANOVA and Tukey’s honest significance difference (HSD)).

Next, we examined the growth rate under normal light conditions (white light, ~30 μmol photons m^−2^ s^−1^). Interestingly, five of the seven *ap* mutants showed faster growth than WT (Figure 2). 

These results were somewhat surprising. We assumed that ROS accumulation is more significant in the *ap* mutant cells than in WT because of the effects of ROS-scavenger treatment on the phototactic sign, which were inhibited in these mutants. ROS accumulation may be hazardous to the cells, and treatment with ROS decreases the ciliary beating frequency of *C. reinhardtii* [22]. Contrary to these expectations, many *ap* strains showed high growth rates, suggesting that increased intracellular ROS levels are not directly responsible for the *ap* phenotype.

### 2.3. Photosynthesis Phenotypes of ap Mutants

**Figure 3:**
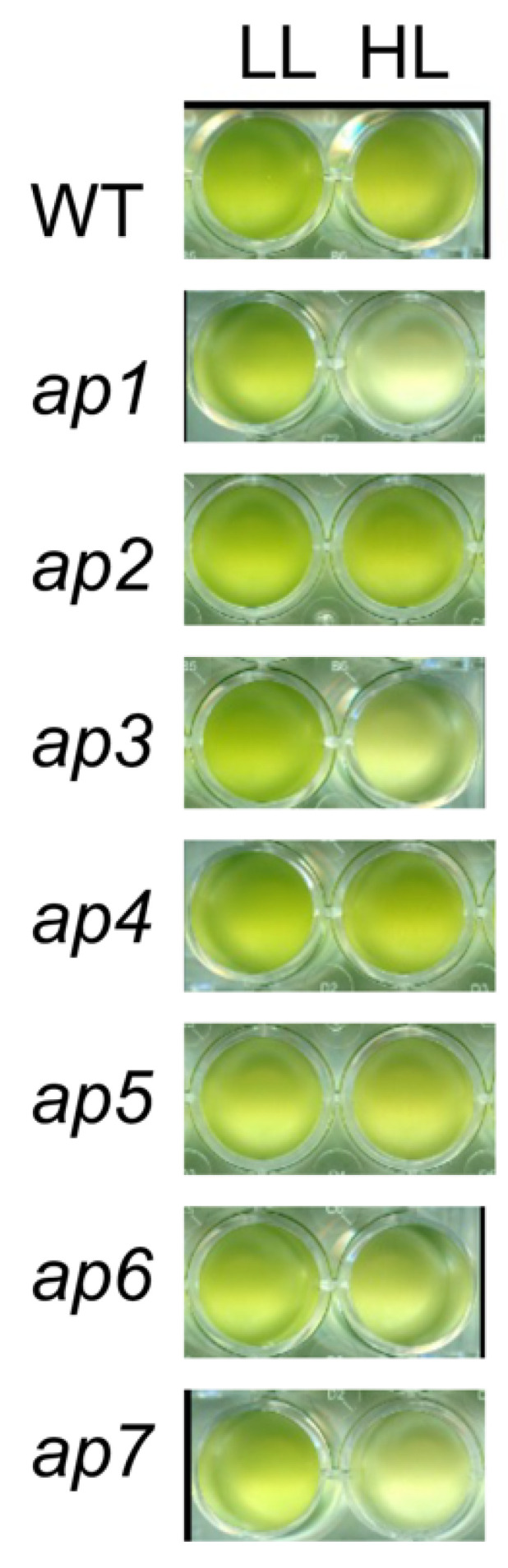
High-light tolerance of *ap* mutants. Cells were grown under low light (LL; white, 50 μmol photons m^−2^ s^−1^) or high light (HL; white, 1000 μmol photons m^−2^ s^−1^) for 18 h. A 70-microliter aliquot was put in a well of a 96-well plate.

We then measured photosynthesis-related parameters such as chlorophyll content, photosynthetic efficiency (φII), and nonphotochemical quenching (NPQ) (Table 2). However, again, there were no significant differences between the strains in each parameter. We also measured survivability under high-light stress conditions. The cells were treated using low light (white, 50 μmol photons m^−2^ s^−1^) or high light (white, 1000 μmol photons m^−2^ s^−1^). The results suggested that *ap1* and *ap7* had a slightly weaker high-light tolerance than WT (Figure 3).

Summarizing the results thus far, phenotypes in *ap1~7* other than positive phototaxis after treatment with DMTU are not necessarily consistent. Most of them grew faster than WT, but not all. There were no significant differences from WT in the photosynthetic parameters. Two of the mutants seemed to exhibit a weaker high-light tolerance than WT.

### 2.4. Detailed Photosynthesis Phenotype Analyses of ap2 and ap7

**Figure 4:**
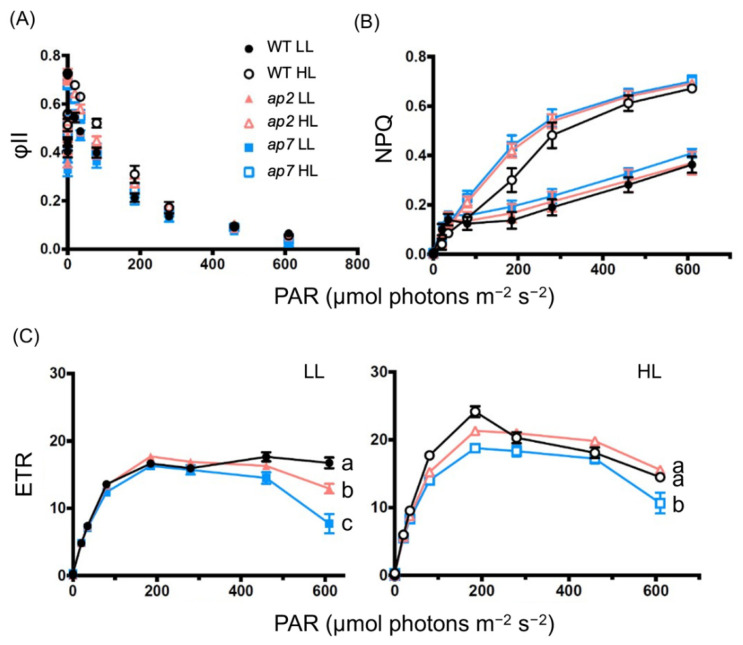
Photosynthetic parameters of *ap2* and *ap7* after treatment with low or high light. (**A**) φII, (**B**) NPQ, and (**C**) ETR of WT, *ap2*, and *ap7* cells after treatment with low light (LL; white, 50 μmol photons m^−2^ s^−1^) or high light (HL; white, 1000 μmol photons m^−2^ s^−1^) (n = 3, mean ± S.E.M.). In (**C**), different letters indicate significant differences (*p* < 0.05, one-way ANOVA and Tukey’s honest significance difference (HSD)). PAR: photosynthetically active radiation.

In the photosynthesis analyses above, we fixed the light conditions to assess the seven mutants simultaneously. For detailed analyses, we selected *ap2* and *ap7*. *Ap2* represents the strains with higher-growth than WT (Figure 2), and *ap7* represents the strains with lower-growth than WT (Figure 3). We treated the cells with low light or high light before the analyses using a pulse amplification modulation and measured the photosynthetic parameters under various light intensities. The values of φII and NPQ change in almost the same manner among the strains (Figure 4A,B). In contrast, both *ap2* and *ap7* showed a lower ETR than WT when pretreated with low light and measured using high light (Figure 4C). Furthermore, only *ap7* showed a lower ETR than WT when pretreated with high light (Figure 4C).

**Figure 5:**
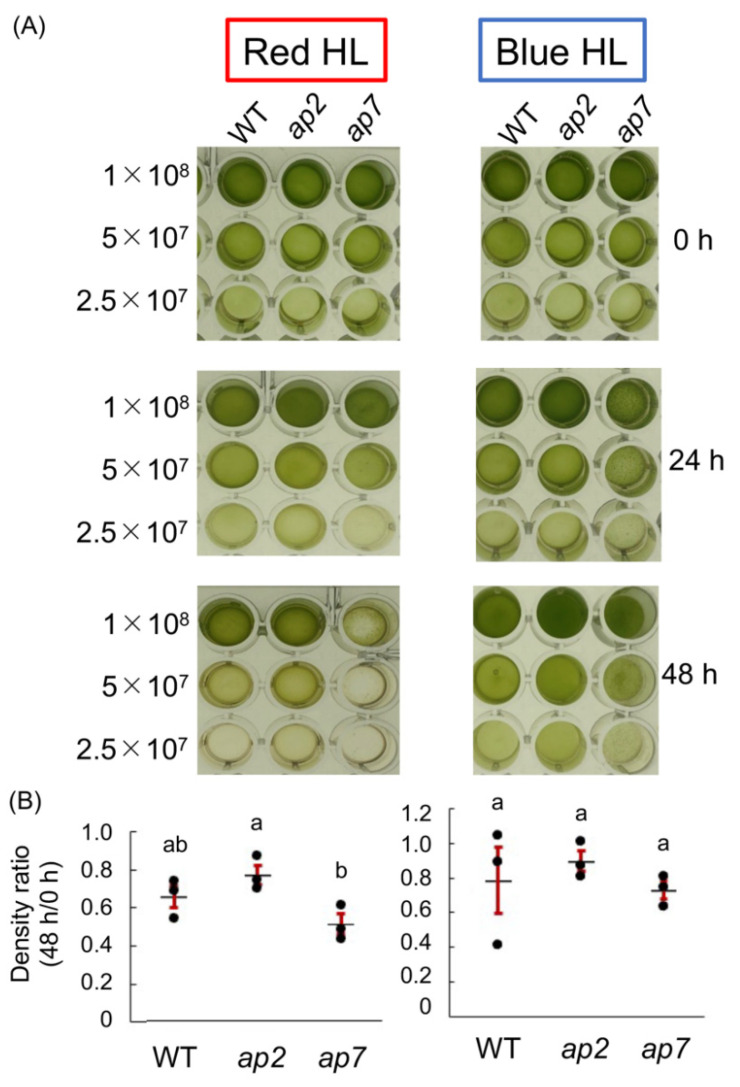
High-light tolerance of *ap2* and *ap7* under red or high blue light. (**A**) Dilution series of WT, *ap2*, and *ap7* cell culture (2.5 × 10^7^–1 × 10^8^ cells/mL) were illuminated with either red high light (left; λ = 640 nm, 800 µmol photons m^−2^ s^−1^) or blue high light (right; λ = 470 nm, 300 µmol photons m^−2^ s^−1^) for two days. (**B**) The density of each well was measured, and the ratio of the value at 48 h per that at 0 h was calculated. Each dot represents the individual result, and mean values (black bars) ± S.E.M. (red bars) (n = 3) are shown. Different letters indicate significant differences (*p* < 0.05, one-way ANOVA and Tukey’s honest significance difference (HSD)).

Next, we examined the high-light tolerance of *ap2* and *ap7* under 700 μmol photons m^−2^ s^−1^ red light or 300 μmol photons m^−2^ s^−1^ blue light. The former conditions induce a slow and the latter conditions cause a fast induction of NPQ [23,24]. Under high red light, similar to the white-light conditions (Figure 3), *ap2* showed a slightly higher and *ap7* showed a slightly lower tolerance than WT (Figure 5A,B). In contrast, under high blue light, *ap2* and *ap7* showed an almost comparable tolerance to WT (Figure 5A,B). With the data showing that mutants exhibit normal NPQ (Figure 4), the difference in high-light tolerance in *ap2* and *ap7* may not be due to NPQ.

## 3. Discussion

In this study, to understand the mechanisms underlying the ROS-dependent regulation of the phototactic sign in *C. reinhardtii*, we isolated new mutants *ap1~7* showing positive phototaxis, even after negative phototaxis by a ROS-scavenger DMTU. However, the causative genes of these mutants could not be traced at this moment because the insertion of the generated vectors did not cause *ap* phenotypes. Instead, we characterized these mutants in various ways.

### 3.1. Phenotypic Discrepancy among ap Mutants

Unexpectedly, the phenotypes of the *ap* mutants were not completely consistent. As for the growth rate, *ap1* and *ap3* were significantly faster, and *ap7* was slightly slower than WT (Figure 2). As for the red high light tolerance, *ap2* was slightly higher and *ap7* was slightly lower than WT (Figure 5). From their common positive-phototaxis phenotype, we assumed that all *ap* mutant cells might accumulate higher levels of ROS, and 75 mM DMTU is not enough to quench them. If all mutants accumulate high ROS levels, some phenotypes would be shared, such as a low tolerance of high light, slow growth, and low ciliary beating frequency [22,25,26], but our data showed that the *ap* mutants did not share such phenotypes commonly. These data suggest that the reasons to show an *ap* phenotype are variable and complex. The function of ROS or ROS scavengers in the phototaxis pathway is still unknown. ROS generation, ROS quenching, and ROS sensing pathways in the cell may be involved in this regulation, and further genetic analysis of *ap* mutants will provide clues to which proteins play essential roles in the ROS-dependent phototactic-sign regulation mechanisms.

### 3.2. Growth Phenotype

Notably, five of the seven *ap* mutants showed a significantly faster growth than WT (Figure 2). It has been reported that *C. reinhardtii* mutants that have acquired resistance to singlet oxygen by gain-of-function mutations that grow faster than wild-type strains under oxidative stress conditions [27]. Such mutations may occur in fast-growing *ap* mutants. Recently, microalgae, including *C. reinhardtii*, have attracted attention as a platform for producing valuable materials [28]. Further analyses to clarify how the *ap* phenotype and fast-growing phenotype are linked will contribute to the application for the improvement in microalgae cultivation.

### 3.3. High-Light Tolerance

In plants and algae, photoprotective mechanisms are activated under high-light conditions, in which ROS-scavenging or excitation-energy-dissipation systems protect cells from photodamage [29,30]. The *C. reinhardtii* mutants lacking these photoprotective systems exhibit a significantly lower tolerance against high light than WT [31,32]. Both the *ap2* and *ap7* strains showed a similar level of tolerance to the wild-type strain under blue high-light conditions, which rapidly induce NPQ through the expression of LHCSR proteins (Figure 5) [23]. Alternatively, under red light conditions, *ap2* and *ap7* showed a slightly higher and lower light tolerance, respectively (Figure 5). The LHCSR protein expression level is lower in red light than in blue light, which leads to lower NPQ [33]. Thus, *ap7* may have defects in photoprotection pathways other than NPQ, correlated with lower ETR under high-light conditions and a lower-growth rate (Figure 2 and Figure 4D).

## 4. Conclusions

We isolated new mutants showing positive phototaxis after the induction of negative phototaxis (*ap1~7*). Though we expected that these mutant cells contain higher ROS levels than WT, five of them showed higher-growth phenotypes without significant morphological, motility-related, or photosynthetic phenotypes. The absence of phenotypes other than phototaxis common to the seven mutants suggests the diversity of the biological parameters involved in the ROS-dependent regulation of phototaxis. Further genetic analyses of the mutants will shed light on the enigmatic ROS-dependent phototaxis regulation.

## 5. Materials and Methods

### 5.1. Cell Culture and Strains

*Chlamydomonas reinhardtii* strains CC-124 (nit1− (nitrate reductase), nit2−, agg1−, and mt− (mating type)) [16] and CC-125 (nit1−, nit2−, and mt+) were used. For the elimination of *agg1* mutation, agg1+ progenies (mt+ and mt−) from the mating of CC-124 and CC-125 were used as wild-type. Cells were grown in a tris-acetate phosphate medium (TAP) medium with aeration at 25 °C on a 12 h/12 h light/dark cycle [34]. For the photosynthetic parameter measurement, cells were collected after culturing in TAP medium, resuspended in high-salt (HS) minimal medium [35], and grown under the same light conditions as above without aeration for one day.

### 5.2. Mutagenesis and Screening for ap Mutants

Wild-type (mt−) cells were mutagenized by the random insertion of pSI103-1 vector (linearized by EcoRI) that confers paromomycin resistance via electroporation (NEPA21, NEPAGENE) [21,36]. After collecting colonies on the selection agar plates, those cells (a mutant library) were subjected to the phototaxis assay after treatment with 75 mM DMTU (Sigma-Aldrich), which strongly induces negative phototaxis [15]. After green light illumination (λ = 525 nm, 30 μmol photons m^−2^ s^−1^), cells showing positive phototaxis were collected. After culturing those mutant candidates for a few days, the same phototaxis assay was repeated. Cells showing positive phototaxis were inoculated onto a TAP agar plate, and grown single colonies were inoculated independently in a TAP medium in a 96-well scale. Cultures were subjected to the phototaxis assay again, and a strain showing positive phototaxis after the DMTU treatment was selected as an *ap* mutant. These assays were conducted against seven mutant libraries.

### 5.3. Cell-Level Phototaxis Assay

Cell-level phototactic motion was tracked based on a previously described method [15] with modifications. Briefly, cells were washed with experimental practical solution (5 mM Hepes (pH 7.4), 0.2 mM EGTA, 1 mM KCl, and 0.3 mM CaCl_2_) with or without treatment with DMTU or *t*-BOOH (FUJIFILM Wako Pure Chemical Corporation, Osaka, Japan) and kept under dim red light for 15 min before the phototaxis assays. The behavior of the cells was observed and video-recorded under a dark-field microscope (BX-53, Olympus) with dim red light under unidirectional illumination using a green light-emission diode (λ = 525 nm, 30 μmol photons m^−2^ s^−1^). The angle (θ) between the light direction and the swimming direction was measured during 1.5 s, following illumination with a green LED for 15 s. Images of swimming cells were auto-tracked using Image Hyper software (Science Eye). The angles were calculated from the cell trajectories.

### 5.4. Ciliary Beating Frequency Measurement

Ciliary beating frequency was measured based on a previously described method [37] with modifications [22]. Briefly, a photodetector was set on the top of a microscope equipped with a dark-field condenser (BX-53; Olympus). Cells were observed under a microscope with a dim red light (λ > 630 nm) to avoid the accumulation of cells caused by phototaxis. The photodetector detected signals derived from cell body vibration, transferred to the computer soundboard, and fast-Fourier transformed using SIGVIEW (SignalLab). Transformed signals were averaged for ~20 s, and the peak value was regarded as the mean ciliary beating frequency.

### 5.5. Cell Density and Cell Size Measurement

Cell culture was mixed with an equal volume of deciliation solution (1 mM CaCl_2_ and 40 mM sodium acetate). Cell density was measured using an automatic cell counter (model R-1, Olympus, or Cell Drop BF, DeNovix). Additionally, the cell size (diameter when a cell is approximated to a sphere) was simultaneously measured using the same cell counter.

### 5.6. Growth Rate Assay

Cells were grown in the TAP medium for three days under 30 μmol photons m^−2^ s^−1^ white light, adjusted to 1 × 10^6^ cells/mL in a fresh TAP medium (day 0), and then grown again under the same light conditions. The cell density of each strain was measured every day as described above.

### 5.7. Chlorophyll Amount Measurement

Cell culture was collected and resuspended in an HS medium, and cell density was measured. Cell suspension (200 μL) and acetone (800 μL) were mixed using a vortex mixer and spun down, and Abs_750_, Abs_663.6_, and Abs_646.6_ of the supernatants were measured. Chlorophyll contents were calculated according to the equations shown in [38].

### 5.8. High-Light Tolerance Assay

A 700-microliter culture in HS medium was placed in a microtube and illuminated with low light (white, 50 μmol photons m^−2^ s^−1^) or high light (white, 1000 μmol photons m^−2^ s^−1^) for 18 h (Figure 2), or with red light (λ = 640 nm, 800 µmol photons m^−2^ s^−1^) or blue light (λ = 470 nm, 300 µmol photons m^−2^ s^−1^) for 48 h (Figure 5). A 70-microliter aliquot was put in a 96-well plate and pictured. The cell density of each well was quantified using Image J in Figure 5.

### 5.9. Photosynthetic Parameter Analyses

The chlorophyll amount of the cell culture in an HS medium was measured, and cell density was adjusted to ensure that the cells contain 2.5 μg/mL chlorophyll. Chlorophyll fluorescence-based photosynthetic analysis was performed as follows. Maximum yields (Fm) were measured under dark conditions (after weak far-red (<5 μmol photons m^−2^ s^−1^) treatment for 30 min using Dual-PAM (WALZ, Germany) for Table 2 or Imaging PAM (WALZ, Germany) for Figure 4. Following the method of [39], the maximum and steady-state fluorescence yields under light (Fm′ and F, respectively) were measured after actinic irradiation at each light intensity for 90 s. The effective PSII quantum yield Y(II) (or φII) was estimated using the equation, Y(II) =  (Fm′ − F)/Fm′. Nonphotochemical quenching capability (NPQ) was estimated using the equation, NPQ =  (Fm −  Fm′)/Fm′. The ETR was estimated using ETR = Y(II) × 0.84 × 0.5 × light intensity.

### 5.10. PCR against Tetrad Progenies

Tetrad progenies were subjected to PCR to determine the presence of the *APHVIII* gene using the method of [40].

## Figures and Tables

**Table 1:** Morphological and motility phenotypes of *ap* mutants.

	WT	*ap1*	*ap2*	*ap3*	*ap4*	*ap5*	*ap6*	*ap7*
Cell size (μm) *	7.5 ± 0.1	7.0 ± 0.1	7.1 ± 0.2	7.3 ± 0.3	7.0 ± 0.1	7.0 ± 0.2	6.9 ± 0.1	7.4 ± 0.1
Ciliary length (μm) **	13.2 ± 1.5	13.9 ± 1.6	13.6 ± 1.7	13.2 ± 1.6	13.7 ± 2.0	13.6 ± 1.6	13.3 ± 1.5	13.5 ± 1.4
Ciliary beating frequency (Hz) *	57.4 ± 0.5	56.0 ± 1.6	57.4 ± 1.1	56.1 ± 2.0	59.0 ± 0.6	53.1 ± 1.1	55.2 ± 1.2	54.7 ± 1.3

No significant difference between any two groups was found in each parameter (*p* > 0.05, one-way ANOVA and Tukey’s honest significance difference (HSD)). * Mean ± S.E.M. of three independent experiments, ** mean ± S.D. of 20 cilia.

**Table 2:** Photosynthesis-related phenotypes of *ap* mutants.

	WT	*ap1*	*ap2*	*ap3*	*ap4*	*ap5*	*ap6*	*ap7*
Chlorophyll (pg/cell) *	0.78 ± 0.10	0.73 ± 0.13	0.82 ± 0.05	0.75 ± 0.07	0.83 ± 0.06	0.81 ± 0.14	0.70 ± 0.05	0.97 ± 0.07
Photosynthetic efficiency (φII) **	0.59 ± 0.01	0.61 ± 0.02	0.59 ± 0.01	0.59 ± 0.02	0.57 ± 0.01	0.58 ± 0.02	0.59 ± 0.02	0.57 ± 0.01
NPQ **	0.30 ± 0.02	0.30 ± 0.02	0.31 ± 0.01	0.29 ± 0.04	0.29 ± 0.03	0.28 ± 0.01	0.29 ± 0.01	0.28 ± 0.01

300 μmol photons m^−2^ s^−1^ white light was irradiated as actinic light. No significant difference between any two groups was found in each parameter (*p* > 0.05, one-way ANOVA and Tukey’s honest significance difference (HSD)). * Mean ± S.E.M. of four independent experiments, ** mean ± S.E.M. of three independent experiments.

## Data Availability

The data presented in this study are available in this article or supplementary material.

## Supplementary Materials

The following are available online at https://www.mdpi.com/article/10.3390/plants10071483/s1, Figure S1: Tetrad analysis to test linkage between phenotype and genotype.
