# Supplementary material for: Characterization of Chlamydomonas reinhardtii Mutants That Exhibit Strong Positive Phototaxis
Source: Plants (Basel). 2021 Jul 20;10(7):1483. doi: 10.3390/plants10071483 (PMC8309459; doi:10.3390/plants10071483)
Supplement: Supplementary file 1 [file plants-10-01483-s001.zip › plants-1285844-supplementary.pdf]

Supplementary Figure

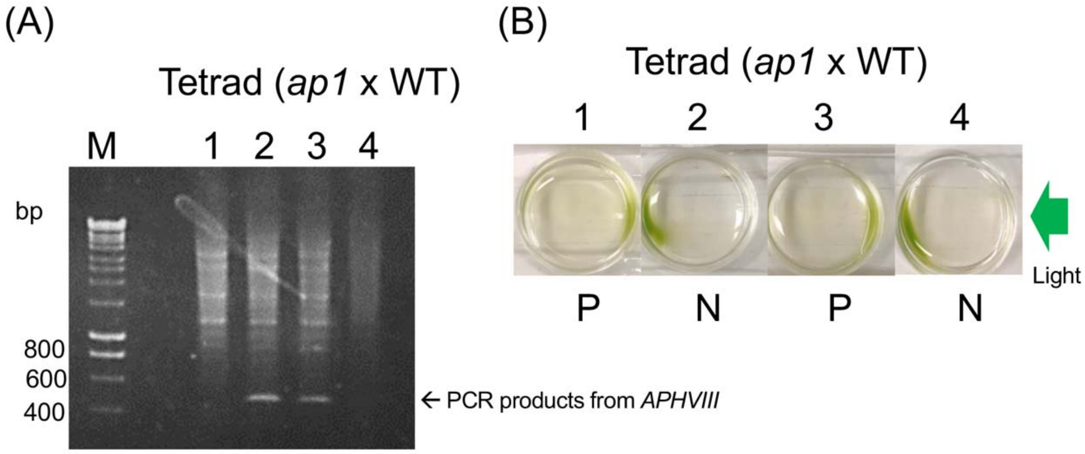

(C)

	ap1		*ap2*		*ap3*		*ap4*		*ap5*		*ap6*		*ap7*	
	APHVIII insertion	Positive phototaxis	*APHVIII* insertion	Positive phototaxis	*APHVIII* insertion	Positive phototaxis	*APHVIII* insertion	Positive phototaxis	*APHVIII* insertion	Positive phototaxis	*APHVIII* insertion	Positive phototaxis	*APHVIII* insertion	Positive phototaxis
Progeny 1	-	+	-	+	-	+	-	+	+	+	-	+	+	-
Progeny 2	+	-	+	-	+	-	+	-	-	-	+	-	+	+
Progeny 3	+	+	+	-	-	-	+	+	-	+	+	-	-	+
Progeny 4	-	-	-	+	+	+	-	-	+	-	-	+	-	-

Figure S1. Tetrad analysis to test linkage between phenotype and genotype. (A) An example of PCR of the tetrad progenies *ap1*× WT. Here, progenies 2 and 3 had *APHIII* insertion. (B) Phototaxis analysis after treatment with 75 mM DMTU for the same tetrad as (A). Progeny 1 and 3 showed positive (P), and 2 and 4 showed negative (N) phototaxis. From the data in (A) and (B), the *ap* phenotype in *ap1* is shown not to be caused by *APHIII* insertion. (C) Representative tetrad analyses of each *ap* mutant. Each tetrad contained a progeny with *APHIII* insertion that did not show positive phototaxis after treatment with DMTU.
